# Supplementary material for: Skilful predictions of the Asian summer monsoon one year ahead
Source: Nat Commun. 2021 Apr 7;12:2094. doi: 10.1038/s41467-021-22299-6 (PMC8027800; doi:10.1038/s41467-021-22299-6)
Supplement: Supplementary file 1 — Supplementary information [file 41467_2021_22299_MOESM1_ESM.pdf]

Supplementary Information on "Skilful predictions of the Asian summer monsoon one year ahead"

Yuhei Takaya, Yu Kosaka, Masahiro Watanabe and Shuhei Maeda

Supplementary Note 1. Local SST-precipitation correlation in summer

In the WNP, local precipitation and SST are negatively correlated in summer (Supplementary Fig. 1), indicating the dominance of local atmospheric forcing on the ocean and thus suggesting a lack of potential predictability from local SST[1,2], which poses a fundamental challenge to climate models in predicting the Indo-WNP monsoon. Although uncoupled atmospheric models have difficulty in reproducing this relationship[1], state-of-the-art climate models, which incorporate an ocean component, can generally reproduce it[2,3] (Supplementary Fig. 1). The success of seasonal and longer predictions of the WNP summer monsoon suggests the dominance of remote rather than local SST influences.

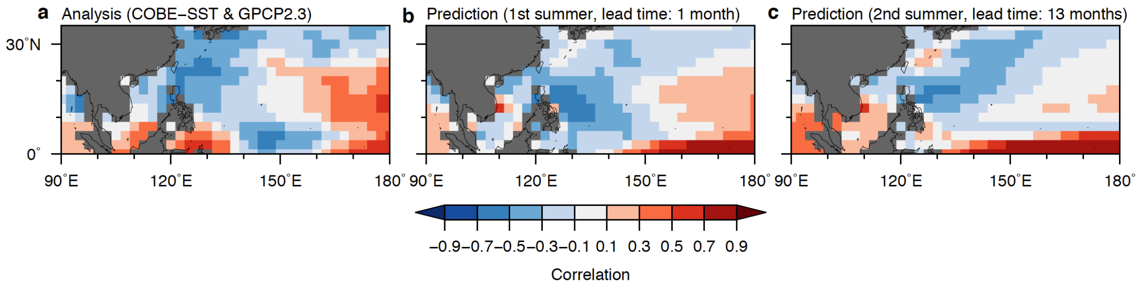

Supplementary Figure 1 | Spatial distribution of local simultaneous SST–precipitation correlations in observations and predictions. Point-wise correlations between JJA-mean precipitation and SST for the 37-year period of 1980–2016. For observations, Centennial in situ Observation-Based Estimates of sea surface temperature (COBE-SST) and precipitation analysis of Global Precipitation Climatology Project (GPCP) version 2.3 (GPCP2.3) are used for this analysis. For predictions, concatenated all-member predictions (1872 seasonal samples of 52 members × 36 years) are used.

Supplementary Note 2. Seasonal dependence of the prediction skill for the NINO3.4 SST and IOB-wide SST, and Dipole Mode Index

The NINO3.4 SST prediction skill decreases as the lead time increases with a relatively large decline across boreal spring, the so-called spring predictability barrier[4]. The IOB SST prediction skill, in contrast, does not decrease but is sustained instead during winter into boreal spring (Supplementary Fig. 2). The prediction skill of the IOB SST peaks in spring, when salient warming of IOB SST occurs in decaying El Niño years[5,6]. The seasonal dependence of the prediction skills obtained in this study is consistent with results in previous studies using two different prediction systems, JMA/MRI-CPS1[3] and JMA/MRI-CPS2[7]. The Dipole Mode Index (DMI), which is defined as the difference between SST anomalies averaged over key western (10°N–10°S, 50°E–70°E) and eastern (Eq. –10°S, 90°E–110°E) regions and represents the Indian Ocean Dipole (IOD), was evaluated[8]. The prediction skill of DMI exhibits the characteristic seasonal dependency with the first peak in boreal autumn, when the IOD matures, and the second peak in boreal spring. The rebound of the IOB SST prediction skill is considered to reflect the delayed influence of ENSO and IOD[6]. This long-lead prediction skill for the IOB SST and its associated remote influences are key for long-lead Asian summer monsoon predictions.

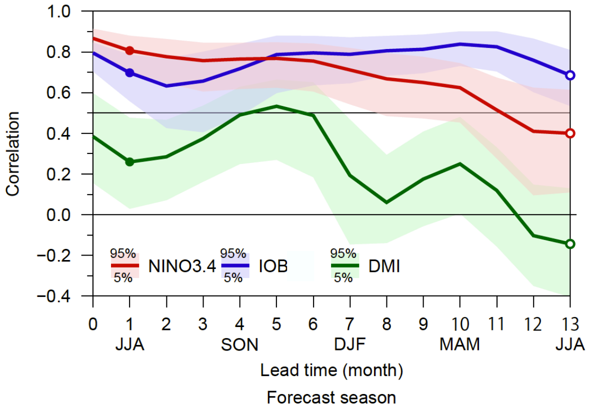

Supplementary Figure 2 | Prediction skills of the NINO3.4 SST, Indian Ocean Basin (IOB) SST and Dipole Mode Index (DMI) as a function of lead time. Correlation coefficient between observations and predictions. Curves indicate correlations with ensemble mean predictions. The first and second summer (June-July-August; JJA) prediction skills are denoted with closed and open circles, respectively. The uncertainty ranges are based on a bootstrap method (10,000 resamplings).

Supplementary Note 3. Relationship between the Indo–western North Pacific summer monsoon and climate conditions in Asia

Accurate representations of teleconnections with the Indo-WNP summer monsoon are key to improving seasonal predictions in the surrounding Asian regions. Supplementary Figure 3 presents the regressed anomalies of several elements of the WNP summer monsoon index with its sign being flipped, in the observations and the first and second summer predictions. Concatenated all-member predictions (1872 season samples of 52 members × 36 years) are used for this analysis. These maps correspond to the conditions typical of El Niño following summers when the WNP summer monsoon tends to be anomalously weak. The observations feature an anomalous lower-tropospheric anticyclone and suppressed rainfall in the tropical WNP, enhanced rainfall over the Indian Ocean and Maritime Continent, and the Pacific−Japan teleconnection pattern[9]. The model successfully reproduces these observed features. This analysis illustrates a strong linkage between the WNP summer monsoon and Asian climate.

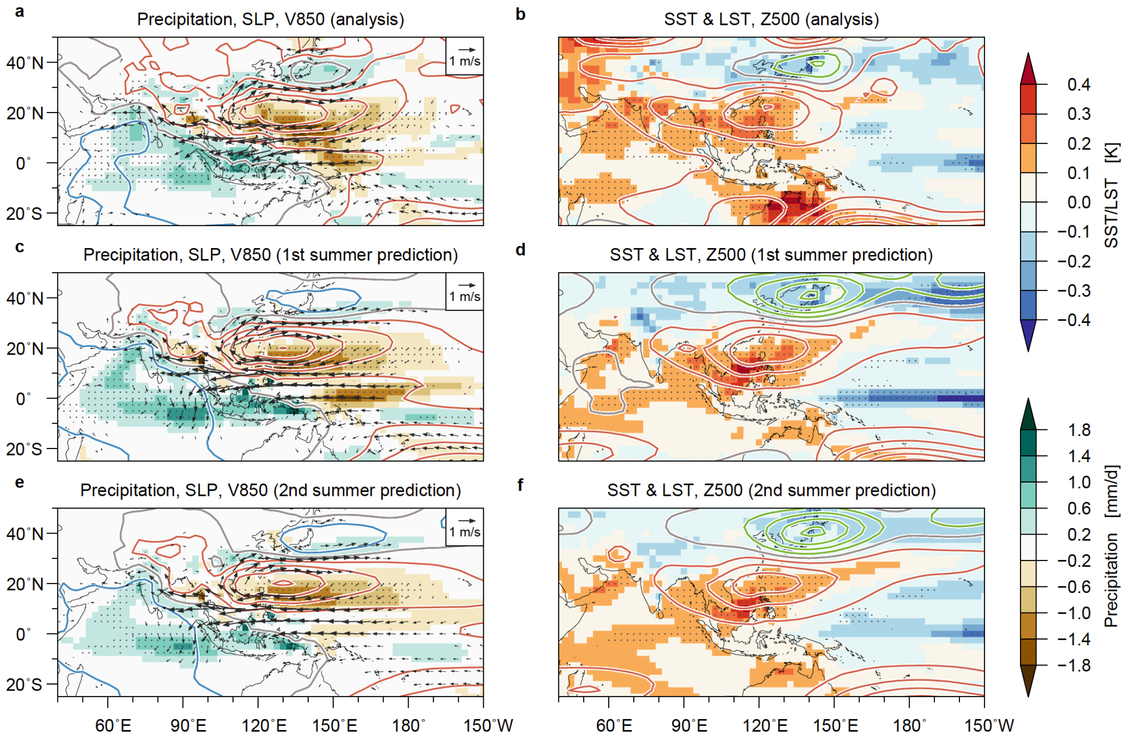

Supplementary Figure 3 | Climate anomalies associated with WNP summer monsoon variability. Summer (JJA) average anomalies of (**a,c,e**) precipitation, sea level pressure (SLP; contours with an interval of 0.2 hPa; red for positive, grey for zero, and blue for negative) and 850-hPa wind (V850). (**b,d,f**) sea surface temperature (SST) and land surface (2-m air) temperature over land (LST) and 500-hPa height (Z500; contours with an interval of 2 m; red for positive, grey for zero, and green for negative) are regressed on the standardized WNP index with its sign being flipped. Results for the (**a**,**b**) observations, (**c**,**d**) first summer prediction and (**e**,**f**) second summer prediction.

Supplementary Note 4. Contributions of the Indo-western Pacific Ocean capacitor and developing and decaying El Niños to the second summer prediction skill

We assess the contributions of the IPOC mode in summers following major El Niño events and concurrent El Niños to the prediction skill for the second summer. To assess the contributions of these years to the prediction skill, we calculate scores by excluding summers of decaying El Niño years (observed NINO3.4 index > 1 std. dev. in preceding November–January; 1983, 1992, 1998, 2003, 2010 and 2016) and developing El Niño years (observed NINO3.4 index > 1 std. dev. in subsequent November−January, consecutive El Niño years excluded; 1982, 1991, 1997, 2002 and 2009). Supplementary Table 1 summarizes the prediction skills for selected indices. Except for NINO3.4 SST and precipitation in the Ganges River Basin, the skills tend to decrease when summers following major El Niños are excluded. In contrast, excluding the summers of developing major El Niños does not affect the skill scores. This indicates that the high model skill in predicting the interannual Indo-WNP monsoon variability in the second summer is explained to a considerable extent by the contribution of decaying El Niño summers but is unlikely to be explained by that of developing El Niño summers. Although the previous study[10] suggested the relation between the IPOC mode and precipitation in the Ganges River Basin, the smaller skill change for precipitation in the Ganges River Basin may impliy additional processes that introduce predictability that are yet to be identified.

Supplementary Table 1 | Correlation skills for the second summer prediction

Cases	WNPM	NINO3.4	IOB*	WNP rain	Ganges rain	Indochina Ts*
All years	0.50	0.41	0.52	0.52	0.48	0.65
No decaying El Niño	0.26	0.50	0.42	0.18	0.50	0.44
No developing El Niño	0.50	0.43	0.55	0.56	0.46	0.67

Correlation skills for all years (1980–2016), all but no decaying El Niño years and all but no developing El Niño years. Indices include the WNP summer monsoon (WNPM), NINO3.4 SST (NINO3.4), IOB SST (IOB), precipitation in the tropical WNP (WNP rain), precipitation in the Ganges River Basin (Ganges rain), and surface air temperature in the Indochina region (Indochina Ts). For indices with asterisks, the correlation skills were computed after linear detrending.

Supplementary Note 5. Assessment of the potential predictability

Potentially predictable components (hereafter, the potential predictability) can be assessed by a so-called perfect model approach[11,12]. In this approach, an ensemble prediction system is assumed to perfectly sample the prediction uncertainty. The potential predictability is assessed by randomly selecting one member as a surrogate observation for each case and then evaluating the prediction skills of the ensemble predictions excluding the selected member. This procedure is repeated 10,000 times to test the statistical significance. It is noted that the perfect model assumption is not always guaranteed due to imperfections in the model physics and uncertainty representation[13,14] but still gives reasonable guidance for the potential predictability[11].

In our analysis, the estimated potential predictability (Supplementary Fig. 4) is generally higher than the actual prediction skills verified against the observations. The spatial distributions of the potential predictability resemble the actual skills in the Indo-WNP (Fig. 2). These results support the highly significant prediction skills of the Asian summer monsoon in some regions. We also investigated the potential predictability after detrending to assess the skill gains from the long-term trend. It is evident that the potential predictability of surface temperature decreases by detrending, in particular over Eurasia, consistent with the previous studies[14]. By contrast, the detrending much less affects the potential predictability for 850-hPa zonal wind and precipitation. These characteristics agree with the actual prediction skill of the model (Fig. 2 and Supplementary Fig. 5).

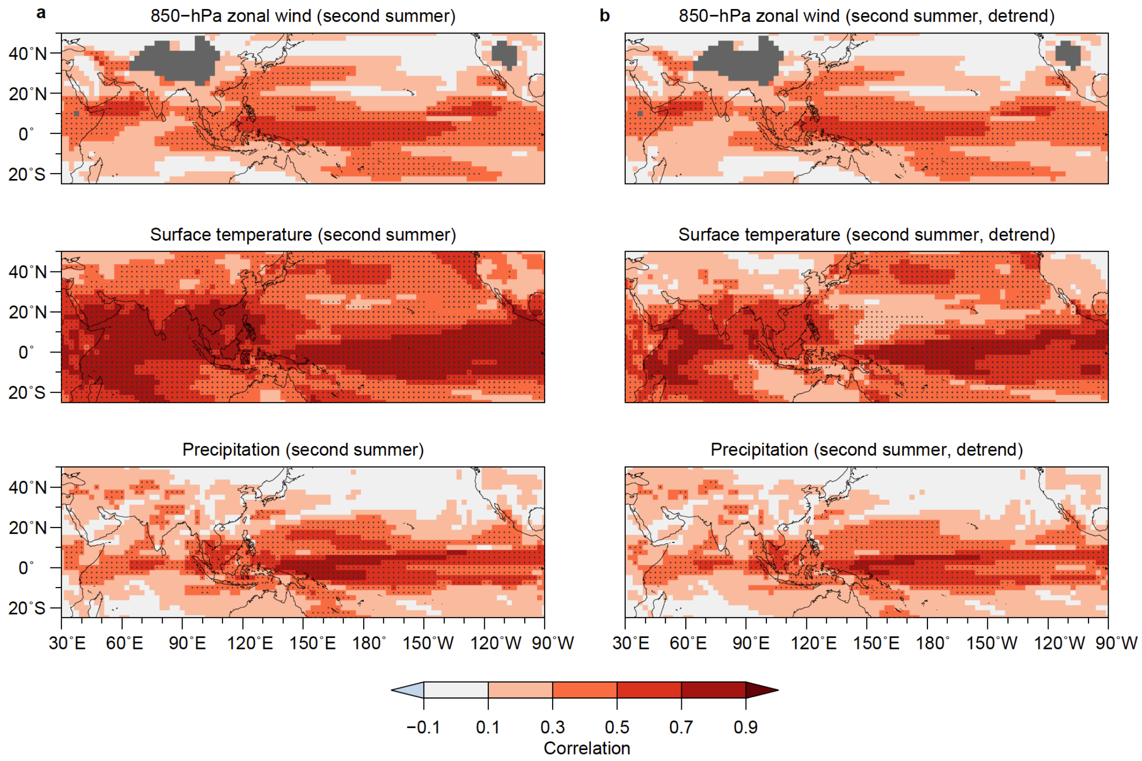

Supplementary Figure 4 | Potential predictability (correlation coefficients) of the second summer predictions (a) without and (b) with detrending. Correlation coefficients between the surrogate observations and ensemble mean predictions for the (top) 850-hPa zonal wind, (middle) 2-m air temperature over land and SST over the ocean, and (bottom) precipitation. Stippled regions are statistically significant at the 5% level according to a bootstrap method (10,000 resamplings).

Supplementary Note 6. Prediction skill after detrending

The prediction skill (correlation scores) is increased due to the long-term trend observed in the verification period[15]. Better representing the trend in the model is important to establish the skilful seasonal prediction, while it is worth evaluating the prediction skill after detrending to confirm whether the model has capability to predict the interannual variability in addition to the trend. Supplementary Figure 5 presents the second-summer prediction skill after detrending. The results (Fig. 2 and Supplementary Fig. 5) indicate that the prediction skill of surface temperature is aided by the trend signal consistent with previous studies[15], whereas that of precipitation and 850-hPa zonal wind is virtually unaffected.

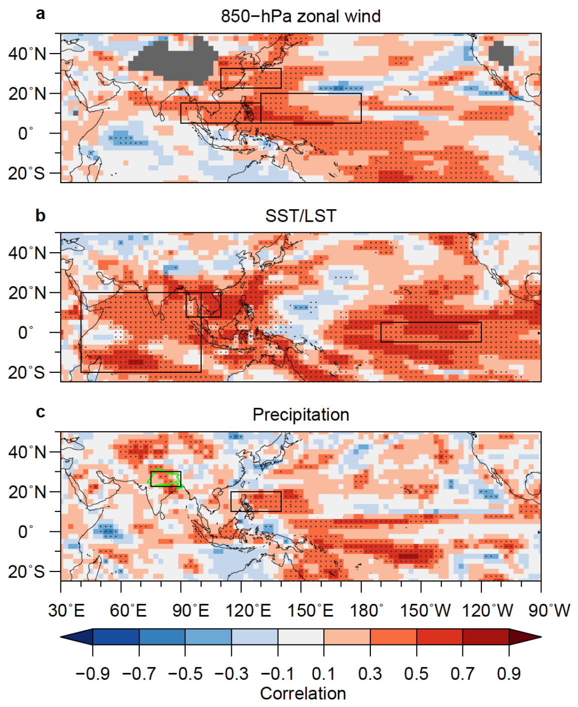

Supplementary Figure 5 | As Fig. 2, but the prediction skill after linear detrending.

Supplementary Note 7. Prediction skill of the Asian monsoon for the first summer

For the first summer, the correlation coefficients of the Asian monsoon indices are generally high ($r > 0.7$, $p < 0.001$), except for the precipitation indices, highlighting the usefulness of operational seasonal predictions of the Asian summer monsoon with a one-month lead time (Supplementary Fig. 6). In contrast to the second summer prediction, the NINO3.4 SST is better predicted than the IOB SST for the first summer (Supplementary Fig. 6), reflecting the seasonal dependence of the prediction skills (Supplementary Fig. 1).

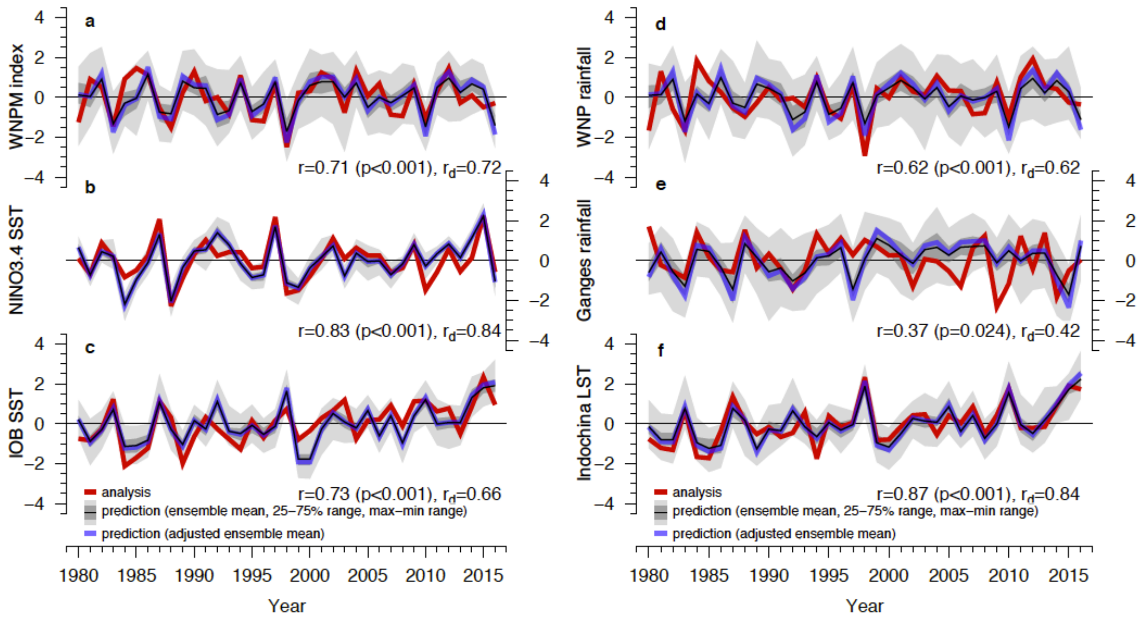

Supplementary Figure 6 | Prediction skill for climate indices in the first summer. Same as Fig. 1, but for the first summer.

Supplementary Note 8. IPOC development along the lifecycle of ENSO

The model's ability to reproduce the IPOC development along the lifecycle of ENSO is evaluated by the lag composite analysis after the major El Niños (Supplementary Fig. 7). The mechanisms of the IPOC evolution after El Niño (see details in the main text) are reasonably reproduced in the model, including the basin-wide warming of IO after El Niño and associated atmospheric conditions[16, 5,6].

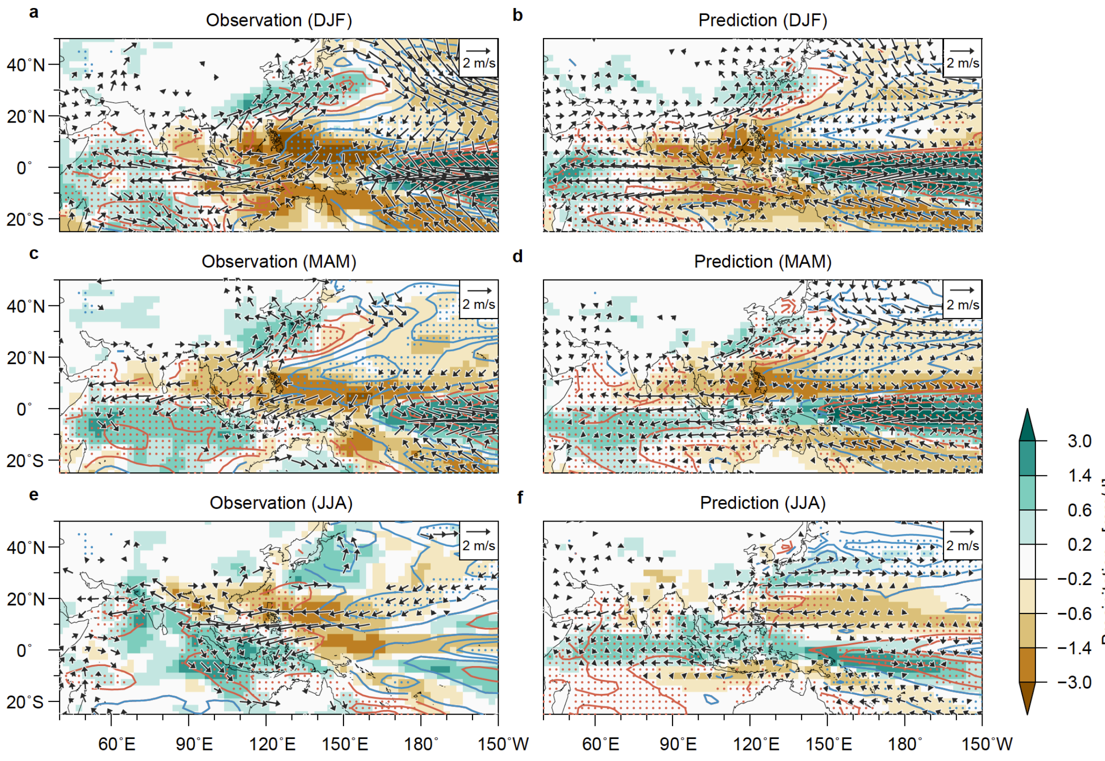

Supplementary Figure 7 | A sequence of IPOC evolution after major El Niño events. Composite anomalies of the (**a**,**c**,**e**) observations and (**b**,**d**,**f**) JMA/MRI-CPS2 prediction for (**a**,**b**) winters (December-January-February; DJF), (**c**,**d**) springs (March-April-May; MAM) and (**e**,**f**) summers (June-July-August; JJA) following the major El Niños. Precipitation (colours), sea surface temperature (contours) and 850-hPa wind (vectors). All the fields are drawn after detrending. Contours are drawn for ±0.2, ±0.4, ±0.8, ±1.6, ±2.4 K. Stippled regions are statistically significant at the 5% level based on a bootstrap method (10,000 resamplings). Vectors are plotted where zonal or meridional wind are statistically significant at the 5% level based on a bootstrap method (10,000 resamplings).

Supplementary Note 9. Indian Ocean and ENSO influences on the WNP summer monsoon and tropical cyclone activity

The Indian Ocean and central-eastern Pacific SSTs have competing effects on the WNP summer monsoon and TC activity. A simple bivariate regression model based on two key SST indices, namely, the NINO3.4 and IOB SSTs, is constructed to evaluate the interbasin SST influences on the WNP summer monsoon (Supplementary Fig. 8a) and TC activity (Supplementary Fig. 8b). The regression model effectively captures the observed year-to-year variability of the WNP summer monsoon (r = 0.60, p < 0.001) and WNP TC activity (r = 0.74, p < 0.001), indicating that the ENSO and Indian Ocean SST are their dominant drivers. The climate model predictions are also in good agreement with the observations (Supplementary Figs. 8a and 8b). The correlation matrix based on the observations is also consistent with the above relationships (Supplementary Fig. 8c).

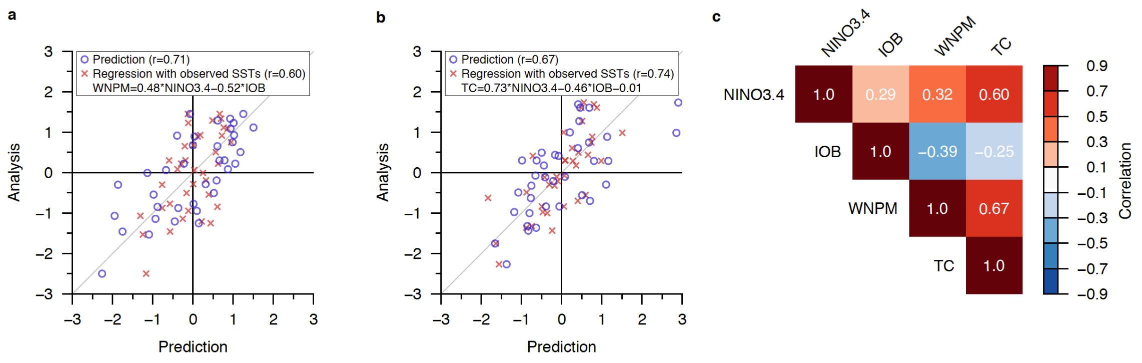

Supplementary Figure 8 | Relationships of the NINO3.4 and IOB SSTs with the WNP summer monsoon and TC activity. Scatterplots of the observed and predicted (**a**) western North Pacific monsoon (WNPM) index and (**b**) tropical cyclone (TC) density. JMA/MRI-CPS2 predictions are denoted by open circles, and bivariate regression model predictions with the NINO3.4 and Indian Ocean basin (IOB) SST indices are denoted as crosses. Regression equations with observed SST indices are shown at the top. (**c**) Correlation matrix for the NINO3.4 SST, IOB SST, western North Pacific (WNP) monsoon and TC density indices. Colours indicate correlation coefficients.

Supplementary References

1. Wang, B. et al. Fundamental challenge in simulation and prediction of summer monsoon rainfall. *Geophys. Res. Lett.* **32**, L15711 (2005).
2. Kumar, A., Chen, M. & Wang, W. Understanding Prediction Skill of Seasonal Mean Precipitation over the Tropics. *J. Clim.* **26**, 5674–5681 (2013).
3. Takaya, Y. et al. Japan Meteorological Agency/Meteorological Research Institute-Coupled Prediction System version 1 (JMA/MRI-CPS1) for operational seasonal forecasting. *Clim. Dyn.* **48**, 313–333 (2017).
4. Webster, P. J. & Yang, S. Monsoon and Enso: Selectively interactive systems. *Q. J. R. Meteorol. Soc.* **118**, 877–926 (1992).
5. Kosaka, Y., Xie, S.-P., Lau, N.-C. & Vecchi, G. A. Origin of seasonal predictability for summer climate over the Northwestern Pacific. *Proc. Natl. Acad. Sci.* **110**, 7574–7579 (2013).
6. Xie, S.-P. et al. Indo-western Pacific ocean capacitor and coherent climate anomalies in post-ENSO summer: A review. *Adv. Atmos. Sci.* **33**, 411–432 (2016).
7. Takaya, Y. et al. Japan Meteorological Agency/Meteorological Research Institute-Coupled Prediction System version 2 (JMA/MRI-CPS2): atmosphere–land–ocean–sea ice coupled prediction system for operational seasonal forecasting. *Clim. Dyn.* **50**, 751–765 (2018).
8. Saji, N. H., B. N. Goswami, P. N. Vinayachandran & T. Yamagata. A dipole mode in the tropical Indian Ocean. *Nature* **401**, 360–363 (1999).
9. Kosaka, Y. & Nakamura, H. Structure and dynamics of the summertime Pacific–Japan teleconnection pattern. *Q. J. R. Meteorol. Soc.* **132**, 2009–2030 (2006).
10. Chowdary, J. S., Patekar, D., Srinivas, G., Gnanaseelan, C. & Parekh, A. Impact of the Indo-Western Pacific Ocean Capacitor mode on South Asian summer monsoon rainfall. *Clim. Dyn.* **53**, 2327–2338 (2019).
11. Rajeevan, M., Unnikrishnan, C. K. & Preethi, B. Evaluation of the ENSEMBLES multi-model seasonal forecasts of Indian summer monsoon variability. *Clim. Dyn.* **38**, 2257–2274 (2012).
12. Dunstone, N. et al. Skilful predictions of the winter North Atlantic Oscillation one year ahead. *Nat. Geosci.* **9**, 809–814 (2016).
13. Scaife, A. A. & Smith, D. A signal-to-noise paradox in climate science. *npj Clim.*

Atmos. Sci. **1**, 28 (2018).

14. Eade, R. et al. Do seasonal-to-decadal climate predictions underestimate the predictability of the real world? *Geophys. Res. Lett.* **41**, 5620–5628 (2014).
15. Doblas-Reyes, F. J., R. Hagedorn, T. N. Palmer, J.-J. Morcrette. Impact of increasing greenhouse gas concentrations in seasonal ensemble forecasts *Geophys. Res. Lett.* **33**, L07708. (2014).
16. Shin, Y. et al. Improved seasonal predictive skill and enhanced predictability of the Asian summer monsoon rainfall following ENSO events in NCEP CFSv2 hindcasts. *Clim. Dyn.* **52**, 3079–3098 (2019).
